# Radiologic simulation of leg length change after double level osteotomy in preoperative surgical planning

**DOI:** 10.1186/s43019-023-00198-y

**Published:** 2023-09-19

**Authors:** Shuntaro Nejima, Ken Kumagai, Shunsuke Yamada, Masaichi Sotozawa, Yutaka Inaba

**Affiliations:** Department of Orthopaedic Surgery, Yokohama City University School of Medicine, 3-9 Fukuura, Kanazawa-ku, Yokohama, 236-0004 Japan

**Keywords:** High tibial osteotomy, Double level osteotomy, Distal femoral osteotomy, Leg length change

## Abstract

**Background:**

To evaluate the expected postoperative total leg length change using preoperative radiographs during surgical planning of four different methods of double level osteotomy (DLO).

**Methods:**

This study included 34 patients (44 knees) who underwent DLO for varus knee osteoarthritis. Surgical planning was performed so that the postoperative weight bearing line ratio was 62.5%. In DLO, lateral closed or medial open wedge distal femoral osteotomy (LCWDFO, MOWDFO) was performed so that the postoperative mechanical lateral distal femoral angle was 85°, and residual deformity was corrected with medial open or lateral closed wedge high tibial osteotomy (MOWHTO, LCWHTO). Pre- and surgical planning X-rays in the one-leg standing position were compared to assess the change in leg length, and the factors affecting it, in the various surgical groups. The proportion of cases in which Δ total leg length was greater than 6 mm (symptomatic change) was investigated.

**Results:**

The mean postoperative total leg length increased significantly with LCWDFO + MOWHTO, MOWDFO + MOWHTO, and MOWDFO + LCWHTO, while it decreased with LCWDFO + LCWHTO. The proportion of cases with a postoperative total leg length change > 6 mm was 72.7%, 2.3%, 100%, and 6.8% in LCWDFO + MOWHTO, LCWDFO + LCWHTO, MOWDFO + MOWHTO, and MOWDFO + LCWHTO, respectively. In addition, the preoperative hip-knee-ankle angle correlated negatively with the postoperative total leg length change in LCWDFO + MOWHTO, MOWDFO + MOWHTO, and MOWDFO + LCWHTO, but not in LCWDFO + LCWHTO.

**Conclusions:**

MOWDFO + MOWHTO had the largest postoperative leg length change and MOWDFO + LCWHTO had the smallest. Symptomatic leg length change (> 6 mm) should be considered in MOWDFO + MOWHTO and LCWDFO + MOWHTO.

## Introduction

Osteotomy around the knee is a well-established surgical option for medial knee osteoarthritis (OA) with varus malalignment [1, 2], and several types of osteotomies have been introduced. Double level osteotomy (DLO), including distal femoral osteotomy (DFO) and high tibial osteotomy (HTO), is one of the procedures for osteoarthritic knees with severe varus deformity [3–5], since excessive overcorrection of a medial proximal tibial angle (MPTA) of greater than 95° following isolated medial open wedge HTO (MOWHTO) leads to greater shear force in the medial cartilage [6] and inferior clinical results [7, 8].

Although the clinical results of osteotomy around the knee are acceptable [9], some complications have been reported [10–12]. One of the problems related to osteotomy around the knee is the postoperative leg length change. Leg length discrepancy can lead to various musculoskeletal problems, such as low back pain [13–15]. However, to the best of our knowledge, only one previous study has reported on leg length change after DLO, including lateral closed wedge DFO (LCWDFO) and MOWHTO [16], although leg length change after MOWHTO and lateral closed wedge HTO (LCWHTO) has been reported in several studies [17–20]. Theoretically, there are four different types of DLOs for varus deformity, based on a combination of the type of osteotomy (medial open wedge and lateral closed wedge) and osteotomy level (femur and tibia). Since leg length change might affect clinical outcomes, surgeons need to consider differences in leg length change with these four different DLO procedures during preoperative planning. However, how leg length changes postoperatively in these four types of DLO has not been well studied.

The purpose of this study was to predict postoperative leg length changes following the four different types of DLO using a virtual surgical planning model. We hypothesized that postoperative total leg length changes vary depending on the DLO procedure performed.

## Materials and methods

This retrospective study included 34 patients (44 knees) who underwent DLO including LCWDFO and MOWHTO for medial knee OA with varus alignment between May 2017 and July 2020. The inclusion criteria were based on our surgical indication: medial compartmental OA of the knee with varus malalignment. Exclusion criteria were symptomatic patellofemoral or lateral compartmental OA, flexion contracture > 15°, and a history of joint infection or inflammatory arthritis. DLO was indicated when the resultant postoperative mechanical medial proximal tibial angle (mMPTA) was expected to be greater than 95° with isolated HTO. Ethical approval for the study was obtained from the institutional review board of our hospital (F211100004). Informed and written consent was obtained from all patients.

### Measurements of joint orientation angles and leg length on radiographs

Anterior–posterior whole-leg radiographs were obtained for preoperative surgical planning with the knee in full extension in the one-leg standing position. The patella was positioned centrally between the femoral condyles. The hip-knee-ankle (HKA) angle, weight bearing line (WBL) ratio, mechanical lateral distal femoral angle (mLDFA), mMPTA, and length of the femur, tibia, and entire leg were measured on the X-rays. These measurements and the surgical planning were performed using Fujifilm OP-A software (Fujifilm Co. Ltd., Tokyo, Japan). The HKA angle was defined as the angle between the femoral and tibial mechanical axes, with a positive value representing valgus alignment. The WBL ratio was defined as the point at which the mechanical axis of the entire leg passed through the tibial plateau. The medial and lateral edges of the tibial plateau were defined as WBL ratios of 0 and 100%, respectively. The mLDFA was defined as the lateral angle between the femoral mechanical axis and the distal femoral joint surface. The mMPTA was defined as the medial angle between the tibial mechanical axis and the proximal tibial joint surface [21]. The length of the femur was defined as the distance between the center of the femoral head and the intercondylar notch. The length of the tibia was defined as the distance between the center of the tibial eminence and the center of the tibial plafond. Total leg length was defined as the distance between the center of the femoral head and the center of the tibial plafond [18, 20].

### Surgical planning of double level osteotomy

**Fig. 1:**
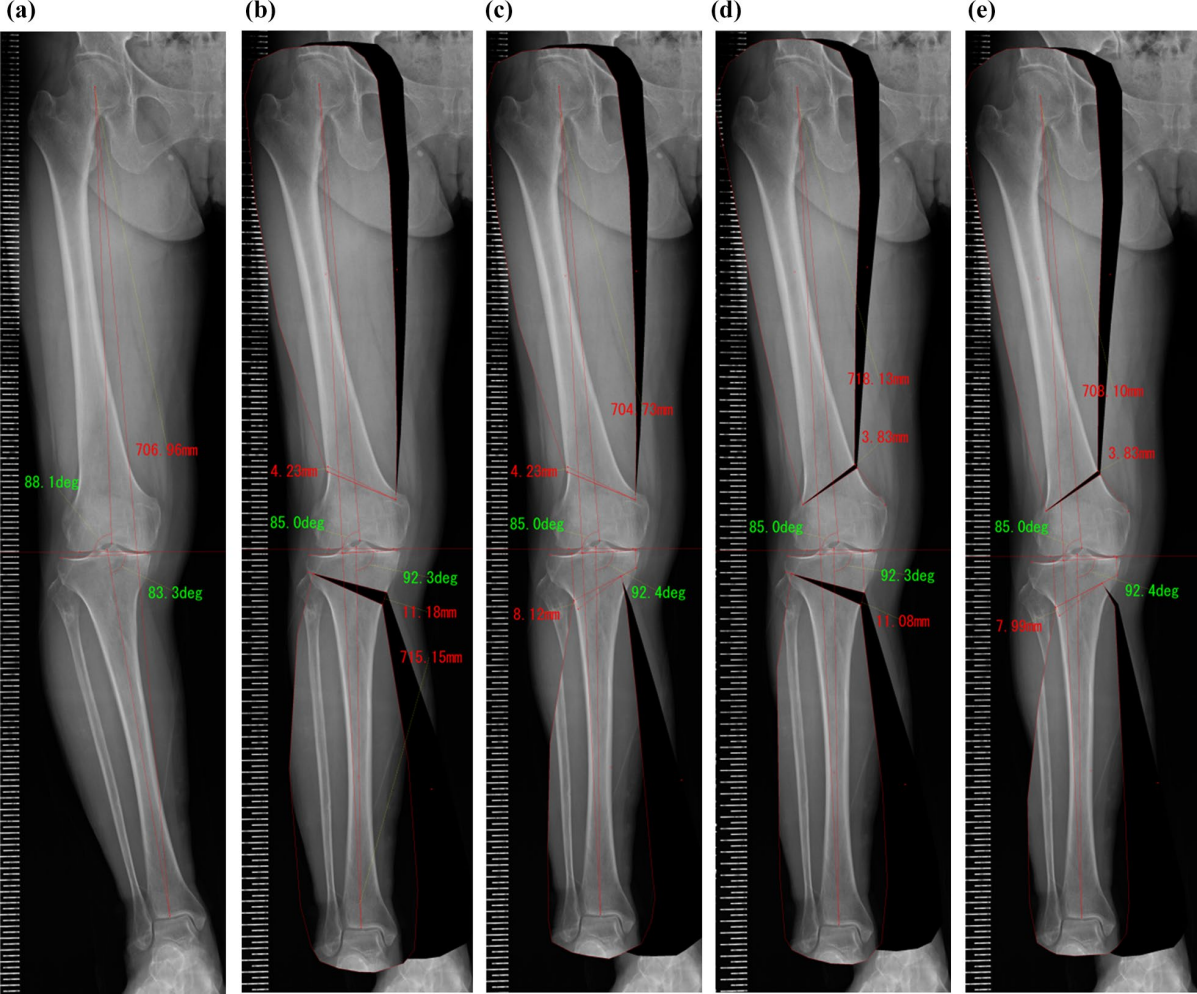
Pre- and post-planning radiographs. **a** Pre-planning radiograph, **b** lateral closed wedge distal femoral osteotomy (LCWDFO) + medial open wedge high tibial osteotomy (MOWHTO), **c** LCWDFO + lateral closed wedge high tibial osteotomy (LCWHTO), **d** medial open wedge distal femoral osteotomy (MOWDFO) + MOWHTO, and **e** MOWDFO + LCWHTO

Surgical planning of DLO was performed to achieve a postoperative WBL ratio of 62.5%. First, DFO was planned. In LCWDFO, a transverse cut was planned 40 mm proximal to the lateral femoral epicondyle and the hinge point was set between the medial femoral condyle and cortex. Another transverse cut was planned proximal to the first transverse cut so that the postoperative mLDFA was 85° [4, 21]. In medial open wedge DFO (MOWDFO), a transverse cut was planned 40 mm proximal to the medial femoral epicondyle and the hinge point was set between the lateral femoral condyle and cortex. Then, the transverse cut was opened so that the postoperative mLDFA was 85°. Residual varus deformity was corrected with HTO. In MOWHTO, a transverse cut was planned 35 mm distal to the medial tibial plateau to the safe zone [22]. A lateral hinge of 5 mm was left. LCWHTO was planned in a hybrid style [23, 24]. A transverse cut was planned 40 mm distal to the lateral tibial plateau to the inflection point of the medial tibial cortex. The hinge point was set to divide the transverse cut line in a 3:1 ratio from the lateral point (Fig. 1). Differences in the length of the femur, tibia, and entire leg between pre- and postoperative radiographs were presented as Δ length of the femur, Δ length of the tibia, and Δ total leg length, respectively. In addition, the proportion of cases in which Δ total leg length was less than − 6 mm and greater than 6 mm was evaluated because a leg length discrepancy of greater than 6 mm was associated with low back symptoms in a previous study [15].

### Statistical analysis

Data are presented as the means and standard deviations. The results were analyzed using IBM SPSS for Windows, version 27.0 (IBM Corporation, Armonk, NY, USA). The paired t-test was used to analyze differences in the lengths of the femur, tibia and entire leg on pre- and postoperative radiographs with each procedure. The proportion of cases with Δ total leg length less than − 6 mm or greater than 6 mm was compared between the four different types of DLO with the Cochran-Q test to determine differences in matched sets of four proportions. If statistical significance was found in comparisons of the four types of DLOs, pairwise comparisons between each of the two procedures were performed and corrected for multiplicity with the Bonferroni test. Moreover, Pearson’s correlation coefficient was used to examine the correlation between preoperative HKA and Δ total leg length in each procedure. A *P* value of less than 0.05 was considered to be statistically significant. Power analysis was performed for the paired t-test (power = 0.9, significance level = 0.05, effect size = 0.5) using G*Power, version 3.1.9.2 (Heinrich-Heine-Universität, Düsseldorf, Germany). An a priori power analysis indicated that 44 knees were needed in this study. Two experienced orthopaedic surgeons (SN, MS) performed surgical planning and measured radiologic parameters. To assess the reliability of measurement of total leg length with each DLO, 20 knees were randomly selected, and intra- and interobserver reliability were assessed by determining the intraclass correlation coefficients (ICCs) for each measurement. Each observer was blinded to the initial findings.

## Results

**Fig. 2:**
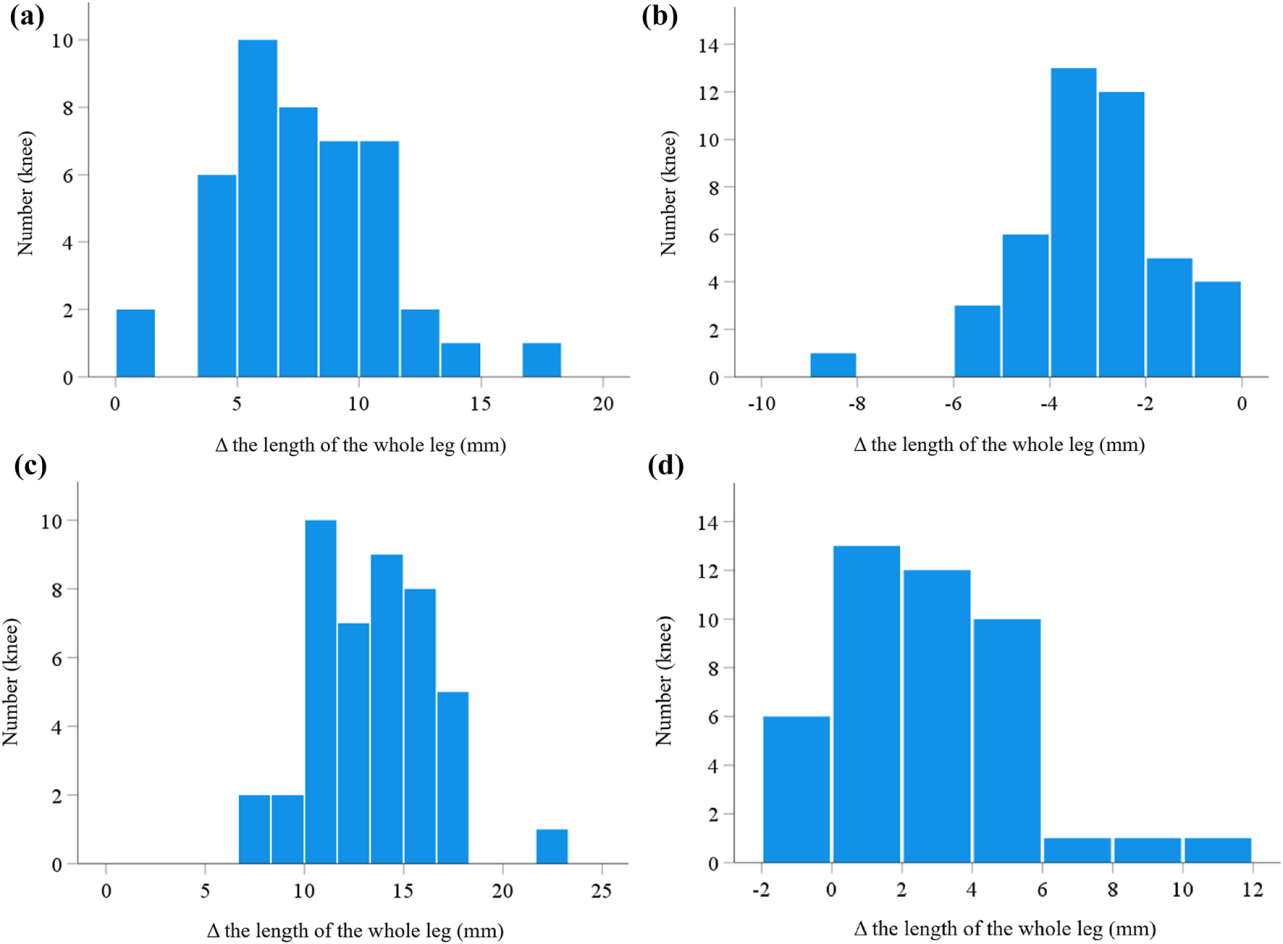
The distribution of change in (Δ) total leg length with each procedure. **a** Lateral closed wedge distal femoral osteotomy (LCWDFO) + medial open wedge high tibial osteotomy (MOWHTO), **b** LCWDFO + lateral closed wedge high tibial osteotomy (LCWHTO), **c** medial open wedge distal femoral osteotomy (MOWDFO) + MOWHTO, and **d** MOWDFO + LCWHTO

Table 1 shows the patients’ demographic and preoperative radiographic data. The postoperative change in leg length is summarized in Table 2. In both the femur and tibia, the closed wedge technique significantly decreased bone length, while the open wedge technique significantly increased bone length. The mean postoperative length of the entire leg increased significantly in LCWDFO + MOWHTO, MOWDFO + MOWHTO, and MOWDFO + LCWHTO, while it decreased in LCWDFO + LCWHTO (Table 2). The distributions of Δ total leg length in each procedure are shown in Fig. 2. The proportion of cases with Δ total leg length less than − 6 mm or greater than 6 mm was higher for LCWDFO + MOWHTO and MOWDFO + MOWHTO, than for LCWDFO + LCWHTO and MOWDFO + LCWHTO (Table 3). In addition, preoperative HKA angle correlated negatively with Δ total leg length in LCWDFO + MOWHTO, MOWDFO + MOWHTO, and MOWDFO + LCWHTO, but not in LCWDFO + LCWHTO (Table 4).

**Table 1:** Patients’ demographic and preoperative radiographic data

Knees, *n*	44
Age, y	62.8 ± 9.7 (41 to 83)
Height, cm	158.4 ± 10.6 (143.5 to 194.3)
Weight, kg	67.1 ± 12.2 (48.6 to 92.9)
Body mass index, kg/m^2^	26.6 ± 3.3 (21.8 to 34.1)
Side, left/right	20/24
Sex, female/male	32/12
Kellgren-Laurence grade 1/2/3/4	0/2/9/33
HKA angle, °	− 11.0 ± 2.5 (− 16.4 to − 6.1)
WBL ratio, %	− 2.0 ± 11.1 (− 29.5 to 15.8)
mLDFA, °	88.9 ± 1.9 (85.9 to 93.3)
mMPTA, °	83.7 ± 2.0 (79.9 to 87.8)
The length of the femur, mm	411.2 ± 37.0 (350.8 to 542.3)
The length of the tibia, mm	340.6 ± 27.5 (303.7 to 422.1)
The length of the whole leg, mm	756.7 ± 64.2 (659.8 to 977.3)

Data are presented as the mean ± standard deviation with the range in parentheses. HKA: hip-knee-ankle; WBL: weight bearing line; mLDFA: mechanical lateral distal femoral angle; mMPTA: mechanical medial proximal tibial angle. The HKA angle with a negative value represents varus alignment. The WBL ratio with a negative value indicates that the mechanical axis of the entire leg passes medial to the medial edge of the tibial plateau

**Table 2:** The postoperative change in leg length

LCWDFO + MOWHTO	
Δ the length of the femur, mm	− 2.7 ± 1.7 (− 6.8 to − 0.3)*
Δ the length of the tibia, mm	6.9 ± 1.6 (3.7 to 10.6)*
Δ the length of the whole leg, mm	7.8 ± 3.3 (0.5 to 17.5)*
LCWDFO + LCWHTO	
Δ the length of the femur, mm	− 2.7 ± 1.8 (− 6.8 to − 0.1)*
Δ the length of the tibia, mm	− 4.0 ± 1.7 (− 8.2 to − 0.9)*
Δ the length of the whole leg, mm	− 3.1 ± 1.7 (− 8.9 to − 0.2)*
MOWDFO + MOWHTO	
Δ the length of the femur, mm	3.0 ± 1.6 (0.2 to 8.2)*
Δ the length of the tibia, mm	6.9 ± 1.6 (4.0 to 11.1)*
Δ the length of the whole leg, mm	13.4 ± 3.1 (7.7 to 23.2)*
MOWDFO + LCWHTO	
Δ the length of the femur, mm	3.0 ± 1.6 (0.3 to 8.2) *
Δ the length of the tibia, mm	− 4.0 ± 1.6 (− 7.8 to − 0.9)*
Δ the length of the whole leg, mm	2.6 ± 2.6 (− 1.6 to 10.6) *

Data are presented as means ± standard deviation with the range in parentheses. LCWDFO: lateral closed wedge distal femoral osteotomy; MOWHTO: medial open wedge high tibial osteotomy; LCWHTO: lateral closed wedge high tibial osteotomy; MOWDFO: medial open wedge distal femoral osteotomy

^*^Significant difference between pre and postoperative length, *P* < 0.001

**Table 3:** The proportion of cases with a postoperative total leg length change > 6 mm

Procedure	The proportion of cases with a postoperative total leg length change > 6 mm
LCWDFO + MOWHTO	72.7% (32/44knees)
LCWDFO + LCWHTO	2.3% (1/44knees)
MOWDFO + MOWHTO	100% (44/44knees)
MOWDFO + LCWHTO	6.8% (3/44knees)
*P* value for all group	< 0.001
*P* value for pairwise comparison	
LCWDFO + MOWHTO vs LCWDFO + LCWHTO	< 0.001
LCWDFO + MOWHTO vs MOWDFO + MOWHTO	n.s
LCWDFO + MOWHTO vs MOWDFO + LCWHTO	< 0.001
LCWDFO + LCWHTO vs MOWDFO + MOWHTO	< 0.001
LCWDFO + LCWHTO vs MOWDFO + LCWHTO	n.s
MOWDFO + MOWHTO vs MOWDFO + LCWHTO	< 0.001

LCWDFO: lateral closed wedge distal femoral osteotomy; MOWHTO: medial open wedge high tibial osteotomy; LCWHTO: lateral closed wedge high tibial osteotomy; MOWDFO: medial open wedge distal femoral osteotomy

**Table 4:** Relationship between preoperative hip-knee-ankle angle and change in total leg length following each procedure

Procedure	*r*	*P*
LCWDFO + MOWHTO	− 0.4	< 0.01
LCWDFO + LCWHTO	0.22	n.s
MOWDFO + MOWHTO	− 0.77	< 0.001
MOWDFO + LCWHTO	− 0.36	< 0.05

LCWDFO: lateral closed wedge distal femoral osteotomy; MOWHTO: medial open wedge high tibial osteotomy; LCWHTO: lateral closed wedge high tibial osteotomy; MOWDFO: medial open wedge distal femoral osteotomy

The intra- and interobserver ICC values were 1.0 and 1.0 for total leg length before planning, 1.0 and 1.0 for that after LCWDFO + MOWHTO, 1.0 and 1.0 for that after LCWDFO + LCWHTO, 1.0 and 1.0 for that after MOWDFO + MOWHTO and 1.0 and 1.0 for that after MOWDFO + LCWHTO, respectively.

## Discussion

The most important finding of this study was that the postoperative change in total leg length varied depending on the DLO procedures. The proportion of cases with postoperative total leg length change with the potential to be symptomatic was greater in LCWDFO + MOWHTO and MOWDFO + MOWHTO, but less in LCWDFO + LCWHTO and MOWDFO + LCWHTO.

In case of a large opening gap with MOWHTO, LCWHTO might be indicated to avoid the risks of patellofemoral joint degeneration, lateral hinge fractures, and delayed bone healing associated with MOWHTO [25–27]. Although the indications for CWDFO versus OWDFO are not clear, a previous study showed that biomechanical stability of the OWDFO with a bone substitute was superior to that of CWDFO [28]. Therefore, in DLO, it might be necessary to select an open or closed technique for the femur and tibia, respectively, depending on the situation. Although a combination of LCWDFO and MOWHTO is the most common procedure [3–5], the present study showed that postoperative total leg length increased in all knees, averaging 7.8 mm in LCWDFO + MOWHTO. A previous study indicated that a leg length discrepancy of greater than 6 mm was related to low back symptoms [15]. In the present study, the proportion of cases with a postoperative total leg length change of greater than 6 mm was 72.7% following LCWDFO + MOWHTO, which is not a negligible proportion and cannot be ignored. Moreover, in MOWDFO + MOWHTO, the postoperative leg length increased by an average of 13.4 mm, and the proportion of cases with a postoperative leg length change of greater than 6 mm was 100%. In contrast, relatively few cases of postoperative leg length change of more than 6 mm were observed for the other two procedures. In particular, in LCWDFO + LCWHTO, despite closed techniques for both the femur and tibia, the postoperative total leg length decreased by an average of only 3.1 mm, and only 2.3% of the knees had a postoperative total leg length change of 6 mm or more. The reason for this, in addition to the influence of whether open or closed techniques are adopted on the femur or tibia, is thought to be that the entire lower limb alignment changes from severe varus to slight valgus following closed wedge procedures, which affects the postoperative total leg length. In MOWDFO + MOWHTO, the femur, tibia, and entire lower limb alignment all change in the direction of a postoperative increase, resulting in a significant increase in the postoperative total leg length. Meanwhile, following LCWDFO + LCWHTO, the entire lower limb alignment changed in the direction of increasing limb length, although both the femur and tibia were shortened, such that the final result was only a small decrease in postoperative total leg length. MOWDFO + LCWHTO resulted in a smaller increase in postoperative total leg length and a smaller proportion of cases of postoperative total leg length change of greater than 6 mm compared to LCWDFO + MOWHTO. This might be due to the study algorithm, according to which the lower limit of mLDFA in DFO is 85° and residual deformity is corrected in the tibia.

In the present study, preoperative HKA angle correlated negatively with the postoperative leg length change in LCWDFO + MOWHTO, MOWDFO + MOWHTO, and MOWDFO + LCWHTO. With these procedures, it is likely that the severe preoperative varus deformity will require more correction, thus increasing postoperative total leg length. However, this was not the case for LCWDFO + LCWHTO. This might be because, as mentioned above, even in cases with a severe preoperative varus deformity, since both the femur and tibia are corrected using a closed technique, the preoperative severe varus deformity is not associated with an increase in postoperative leg length.

Iseki et al. [16] reported that after LCWDFO + MOWHTO, the difference compared to the preoperative length was not significant, although total leg length increased by an average of 2.3 mm. Meanwhile, surgical planning in the present study showed a significant increase in the postoperative total leg length by an average of 7.8 mm following LCWDFO + MOWHTO. This might be due to the different postoperative target alignment in our study versus that in their study. In the present study, the target alignment was planned with a WBL ratio of 62.5%, whereas in their study, the planned WBL ratio was 52–55%. Since both studies set the lower limit of mLDFA at 85° in LCWDFO, it is likely that the correction in MOWHTO in the present study was greater than in their study, leading to an increase in postoperative total leg length.

Although this study included patients who underwent DLO, unicompartmental knee arthroplasty (UKA) can also be indicated for medial knee OA with varus alignment. Generally, osteotomy is indicated for young and active patients. In addition, previous systematic reviews found that UKA was superior in pain and complications, while HTO was superior in knee range of motion [29, 30]. Although both procedures are satisfactory operative treatment options, surgeons should consider the appropriate procedure for each patient.

The clinical relevance of the present study is that in DLO, postoperative Δ total leg length was dependent on the surgical procedure. In particular, LCWDFO + MOWHTO and MOWDFO + MOWHTO are likely to result in a postoperative leg length change that can be symptomatic. In addition, in LCWDFO + MOWHTO, MOWDFO + MOWHTO, and MOWDFO + LCWHTO, the more severe the preoperative varus deformity, the greater the increase in postoperative total leg length, although this is not true with LCWDFO + LCWHTO. Thus, leg length should be considered in the surgical planning of DLO, and the types of DLO should be selected in consideration of the procedure in the contralateral knee. In addition, the patient should be informed preoperatively of the risk of postoperative leg length discrepancy.

There are a few limitations to this study. First, this study was based on the simulation of preoperative radiological planning without assessing postoperative leg length change in patients who underwent DLO. A postoperative total leg length change should be different according to target alignment, amount of correction angle, soft tissue laxity, as expressed by joint line convergence angle, and surgical technique. Thus, the proportion of cases with a postoperative leg length change of greater than 6 mm should be limited to the protocol of this study. Further clinical study will be required to verify the results of the present study. Second, the side-to-side difference in total leg length was not evaluated.

## Conclusions

MOWDFO + MOWHTO had the largest postoperative leg length change and MOWDFO + LCWHTO had the smallest. Symptomatic leg length change (> 6 mm) should be considered in MOWDFO + MOWHTO and LCWDFO + MOWHTO.

## Data Availability

The data and materials are available upon request.

## Abbreviations

OA: Osteoarthritis
DLO: Double level osteotomy
DFO: Distal femoral osteotomy
HTO: High tibial osteotomy
MPTA: Medial proximal tibial angle
MOWHTO: Medial open wedge high tibial osteotomy
LCWDFO: Lateral closed wedge distal femoral osteotomy
LCWHTO: Lateral closed wedge high tibial osteotomy
mMPTA: Mechanical medial proximal tibial angle
HKA: Hip-knee-ankle
WBL: Weight bearing line
mLDFA: Mechanical lateral distal femoral angle
MOWDFO: Medial open wedge distal femoral osteotomy
UKA: Unicompartmental knee arthroplasty
